# Functional brain changes using electroencephalography after a 24-week multidomain intervention program to prevent dementia

**DOI:** 10.3389/fnagi.2022.892590

**Published:** 2022-10-12

**Authors:** Hee Kyung Park, Seong Hye Choi, SeonMyeong Kim, Ukeob Park, Seung Wan Kang, Jee Hyang Jeong, So Young Moon, Chang Hyung Hong, Hong-Sun Song, Buong-O Chun, Sun Min Lee, Muncheong Choi, Kyung Won Park, Byeong C. Kim, Soo Hyun Cho, Hae Ri Na, Yoo Kyoung Park

**Affiliations:** ^1^Department of Neurology, Ewha Womans University School of Medicine, Seoul, South Korea; ^2^Department of Mental Health Care of Older People, Division of Psychiatry, University College London, London, United Kingdom; ^3^Department of Neurology, Inha University School of Medicine, Incheon, South Korea; ^4^iMediSync Inc., Seoul, South Korea; ^5^Data Center for Korean EEG, College of Nursing, Seoul National University, Seoul, South Korea; ^6^Department of Neurology, Ajou University School of Medicine, Suwon, South Korea; ^7^Department of Psychiatry, Ajou University School of Medicine, Suwon, South Korea; ^8^Department of Sports Sciences, Korea Institute of Sports Science, Seoul, South Korea; ^9^Graduate School of Physical Education, College of Arts and Physical Education, Myongji University, Seoul, South Korea; ^10^Department of Sports and Health Science, Shinhan University, Uijeongbu-si, South Korea; ^11^Department of Neurology, Dong-A University College of Medicine, Busan, South Korea; ^12^Department of Neurology, Chonnam National University Medical School and Hospital, Gwangju, South Korea; ^13^Department of Neurology, Bobath Memorial Hospital, Seongnam, South Korea; ^14^Department of Medical Nutrition, Graduate School of East-West Medical Nutrition, Kyung Hee University, Yongin, South Korea; ^15^Department of Food Innovation and Health, Graduate School of East-West Medical Nutrition, Kyung Hee University, Yongin, South Korea

**Keywords:** cognitive impairment, biomarkers, quantitative electroencephalography, dementia, multidomain intervention

## Abstract

Quantitative electroencephalography (QEEG) has proven useful in predicting the response to various treatments, but, until now, no study has investigated changes in functional connectivity using QEEG following a lifestyle intervention program. We aimed to investigate neurophysiological changes in QEEG after a 24-week multidomain lifestyle intervention program in the SoUth Korean study to PrEvent cognitive impaiRment and protect BRAIN health through lifestyle intervention in at-risk elderly people (SUPERBRAIN). Participants without dementia and with at least one modifiable dementia risk factor, aged 60–79 years, were randomly assigned to the facility-based multidomain intervention (FMI) (*n* = 51), the home-based multidomain intervention (HMI) (*n* = 51), and the control group (*n* = 50). The analysis of this study included data from 44, 49, and 34 participants who underwent EEG at baseline and at the end of the study in the FMI, HMI, and control groups, respectively. The spectrum power and power ratio of EEG were calculated. Source cortical current density and functional connectivity were estimated by standardized low-resolution brain electromagnetic tomography. Participants who received the intervention showed increases in the power of the beta1 and beta3 bands and in the imaginary part of coherence of the alpha1 band compared to the control group. Decreases in the characteristic path lengths of the alpha1 band in the right supramarginal gyrus and right rostral middle frontal cortex were observed in those who received the intervention. This study showed positive biological changes, including increased functional connectivity and higher global efficiency in QEEG after a multidomain lifestyle intervention.

**Clinical trial registration:**

[https://clinicaltrials.gov/ct2/show/NCT03980392] identifier [NCT03980392].

## Introduction

Quantitative electroencephalography (QEEG) is a real-time, low-cost, non-invasive functional marker that reflects synaptic activity in the brain (Smailovic and Jelic, 2019). In Alzheimer’s disease (AD), the hallmarks of EEG abnormalities include a shift of the power spectrum, consisting of an increase in delta power and theta power and a parallel decrease in alpha power and beta power, and along with a decrease in the coherence of fast rhythms (Jeong, 2004). Increased theta power and decreased beta power, the earliest changes in patients with AD, were also shown in amnestic mild cognitive impairment (aMCI) (Roh et al., 2011; Han et al., 2021). People with MCI who progressed to AD had lower alpha relative power and absolute power than those with stable MCI, indicating that resting state alpha activity declines gradually as cognitive functions are progressively impaired (Lejko et al., 2020). One important feature of QEEG in AD and MCI is the loss of small-world architecture (Stam et al., 2007; Zeng et al., 2015). In addition, QEEG is correlated with scores on the Mini Mental State Examination (MMSE) (Engels et al., 2015), fluid biomarkers (Jelic et al., 1998; Smailovic et al., 2018), and structural changes (Babiloni et al., 2009, 2013, 2015) in AD.

QEEG has proven useful in predicting the response to treatment. Previous studies have demonstrated that delta and theta activity were decreased by the use of acetylcholinesterase inhibitors (Kogan et al., 2001; Adler et al., 2004; Gianotti et al., 2008). Although several studies reported changes in QEEG after a single-domain intervention program such as cognitive training or physical exercise (Huang et al., 2016; Gandelman-Marton et al., 2017), the intervention studies that assessed biological changes using EEG had small sample sizes. Furthermore, until now, no study has investigated changes in functional connectivity using QEEG following a multidomain lifestyle intervention program.

The Finnish Geriatric Intervention Study to Prevent Cognitive Impairment and Disability (FINGER) study is representative of studies that aim to investigate the efficacy of a multidomain intervention program in preventing dementia. This 2-year, double-blind, randomized controlled trial found that multidomain interventions could improve cognitive function in at-risk older adults (Ngandu et al., 2015). This led to a major shift in the focus of dementia research to interventions with modifiable risk factors. However, the Multidomain Alzheimer Preventive Trial, a 3-year, randomized, placebo-controlled trial, failed to show prevention of cognitive decline (Andrieu et al., 2017). Furthermore, there were no differences in structural brain imaging between the intervention group and the control group in the FINGER study (Stephen et al., 2019), suggesting that this multidomain intervention program produced no demonstrable biological effects. The mixed results of a multidomain intervention studies with respect to dementia prevention point to the need for further investigation of the biological effects of such programs.

We demonstrated that the multidomain lifestyle intervention program designed to be suitable for older Korean individuals was feasible and effective in the SoUth Korean study to PrEvent cognitive impaiRment and protect BRAIN health through lifestyle intervention in an at-risk elderly people (SUPERBRAIN) (Park et al., 2020; Moon et al., 2021). In this study, we aimed to investigate the impact of a 24-week multidomain lifestyle intervention on functional brain changes in QEEG using data from the SUPERBRAIN. We hypothesized that there would be a difference in the electrophysiologic changes in QEEG from baseline to the study end between the intervention and control groups.

## Materials and methods

### Study population

A total of 152 participants aged 60–79 years from eight medical centers were enrolled in the SUPERBRAIN study, a 24-week, multicenter, outcome assessor-blinded, randomized controlled trial. Details of the study protocol have been described previously (Park et al., 2020).

The inclusion criteria were as follows: (1) 60–79 years of age; (2) at least one modifiable risk factor for dementia such as hypertension, diabetes mellitus (DM), dyslipidemia, smoking, obesity, abdominal obesity, metabolic syndrome, low level of education (≤9 years), social inactivity, and physical inactivity; (3) *z* score on the Korean MMSE (K-MMSE) above –1.5; (4) Korean Instrumental Activities of Daily Living score <0.4 (Chin et al., 2018); (5) ability to read and write; and (6) presence of a reliable informant. Participants were excluded if they had major psychiatric illnesses, dementia, substantial cognitive decline, other neurodegenerative diseases, cancer over the past 5 years, serious or unstable symptomatic cardiovascular diseases, stent insertion in coronary vessels within the previous year, and other serious medical conditions. In addition, if subjects were uncooperative or not able to take part in the intervention programs, they were excluded from this study.

**FIGURE 1:**
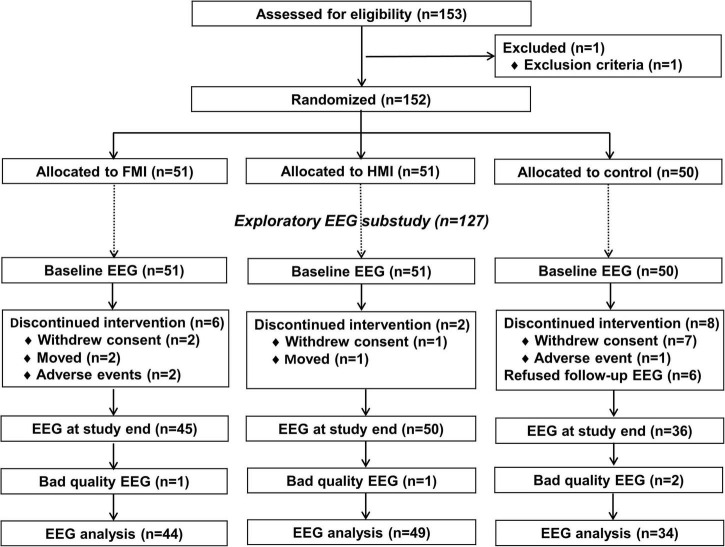
Diagram depicting the exploratory EEG substudy in the SUPERBRAIN trial. FMI, facility-based multidomain intervention; HMI, home-based multidomain intervention; EEG, electroencephalography.

Figure 1 shows a flow chart of the study. All participants were randomly assigned to three groups, consisting of the facility-based multidomain intervention (FMI, *n* = 51), home-based multidomain intervention (HMI, *n* = 51), and control group (*n* = 50), in a 1:1:1: ratio using a permuted block randomization method, with block sizes of three and six, through SAS macro programming, and was stratified by the participating center. The allocation sequence was known only to the independent statistical specialist. Cognitive outcome assessors remained blind to the assigned groups; they were not involved in the intervention activities. Participants were instructed not to discuss their study involvement with the outcome assessor. A total of 45, 49, and 42 participants completed the study from the FMI, HMI, and control groups, respectively. Among them, EEG was performed at baseline and at the end of the study in 45, 49, and 36 participants in the FMI, HMI, and control groups, respectively. In this study, we included one participant in the HMI group who underwent follow-up EEG at the early termination of the study. Due to bad EEG quality, 1, 1, and 2 participants in the FMI, HMI, and control groups, respectively, were excluded from the EEG analysis. Finally, the analysis of this study included data of 44, 49, and 34 participants in the FMI, HMI, and control groups, respectively (Figure 1). Because EEG signals are very sensitive, measurements can contain various noise signals. We considered different types of noise (vertical electrooculogram, horizontal electrooculogram, electromyography, body shaking, swallowing, etc.). Nevertheless, if noise appears strongly throughout the data, it may lose its meaning as an EEG signal. Therefore, the criteria for excluded data were determined after checking the signal quality of the raw data. More information can be found in the Supplementary Figure 1.

No differences in age, sex, education level, diagnosis of MCI, vascular risk factors, depression scale, and cognition were found between participants whose EEG data were analyzed (*n* = 127) and those who were excluded (*n* = 25; Supplementary Table 1).

### Intervention and evaluation

The FMI and the HMI intervention groups received intervention consisting of five components (Supplementary Figure 2). Management of metabolic and vascular risk factors consisted of six sessions with a research nurse, including two sessions with an added study physician. At each session, blood pressure, height, weight, waist circumference, smoking, and alcohol drinking were assessed. Participants were given information about their vascular risk factors and were offered prescriptions if necessary. Cognitive training consisted of computerized cognitive training (in-house program) and workbooks targeting various cognitive domains, especially memory, for 50 min twice weekly. The physical exercise program, which consisted of aerobic exercise, muscle strengthening activities, balance training, and exercise to enhance flexibility, was provided for 60 min three times weekly by trained exercise professionals. Based on the Mediterranean-Dietary Approaches to Stop Hypertension diet Intervention for Neurodegenerative Delay diet, called the MIND diet, the nutritional intervention was designed by nutritionists to be familiar to older Koreans. Three individual sessions (tailored diet for the participant) and seven group sessions (education on the MIND diet, practical exercises via a cooking lessons) were provided by study nutritionists. The motivation enhancement program included four 50-min group sessions to educate the importance of lifestyle changes for the prevention of dementia. Participants were also encouraged to engage with the intervention program by watching pop-up pre-recorded video messages from family members. Achievement in the motivational program was assessed by participants themselves. The waitlist control group received a booklet that included lifestyle guidelines to prevent dementia. The multidomain intervention program was provided to the control group after the end of the study.

Demographic and clinical factors evaluated included age, sex, education, obesity, abdominal obesity, physical activity, social activity, apolipoprotein E genotype, and family history of dementia. Medical history was assessed, including hypertension, DM, dyslipidemia, cardiac disease, history of stroke, and MCI. Current smoking and current alcohol consumption were also evaluated. The K-MMSE and the Repeatable Battery for the Assessment of Neuropsychological Status (RBANS) were used as neuropsychological tests both at baseline and at the end of the study. Blood pressure, abdominal circumference, body mass index, total cholesterol, triglycerides, low-density lipoprotein (LDL) cholesterol, high-density lipoprotein (HDL) cholesterol, glucose, and hemoglobin A1c were measured at baseline and at the end of the study.

### Electroencephalography analysis

Resting-state EEG was recorded over a 3-min period with eyes open and another 3-min period with eyes closed using 19 electrodes based on the international 10–20 system (Fp1, Fp2, F7, F3, Fz, F4, F8, T7, C3, Cz, C4, T8, P7, P3, Pz, P4, P8, O1, and O2) at baseline and at the end of the study. The EEG signal was digitized after it was filtered with a band pass of 0.5–70 Hz.

Artifacts were removed in two steps. First, non-stationary bad epochs were totally rejected. Second, stationary bad components related to eye movement, electrocardiography, or electromyography were removed by adaptive mixture-independent component analysis (AMICA). At the sensor level, the absolute power of EEG, the square of the amplitudes, was calculated using fast Fourier transform (FFT) spectral analysis in each of the following eight frequency bands: delta (1–4 Hz); theta (4–8 Hz); alpha1 (8–10 Hz); alpha2 (10–12 Hz); beta1 (12–15 Hz); beta2 (15–20 Hz); beta3 (20–30 Hz); and gamma (30–45 Hz). To calculate the relative power, the absolute power of each frequency band was divided by the total power. The band power ratios, including the theta-to-alpha (TAR), delta-to-alpha (DAR), theta-to-beta (TBR), and theta-to-beta2 (TB2R) ratios, were calculated. In the source-level analysis, the standardized low-resolution brain electromagnetic tomography (sLORETA) was used with 68 regions of interest (ROIs) based on the Desikan-Killiany atlas. The imaginary part of coherence (iCoh) was calculated as functional connectivity among 68 ROIs at eight frequencies (Nolte et al., 2004). Every EEG feature was analyzed by the cloud-based QEEG analysis platform, iSyncBrain^®^ (iMediSync Inc., Republic of Korea^1^).

An undirected binary network was constructed using the iCoh matrix of each frequency band taking the density of the network (25%) into consideration (Hassan et al., 2014; Liu et al., 2017). Measurements of network nodes and edges, defined as the 68 ROIs, consisted of node degree, clustering coefficient, characteristic path length, and small-worldness (Xia et al., 2013). In this study, the characteristic path length was used to measure functional integration (Rubinov and Sporns, 2010).

### Ethical issues

Written informed consent was obtained from all participants by a study physician. The study protocol was approved by Inha University Hospital Institutional Review Board (IRB)(INHAUH-2018-11-022), Ewha Womans University Mokdong Hospital IRB (EUMC-2019-04-013), Ajou University Hospital IRB (AJIRB-BMR-SUR-19-070 and AJIRB-BMR-SUR-19-077), Dong-A University Hospital IRB (DAUHIRB-19-078), and Chonnam National University IRB (CNUH-2019-139) before participant enrollment in the study. The trial has been registered with ClinicalTrials.gov (NCT03980392). The study was carried out in accordance with the International Conference on Harmonization Good Clinical Practice Guideline.

### Statistical analysis

The modified-intention-to-treat population were used in the analysis. The chi-square test for categorical variables and one-way analysis of variance for continuous variables were used to compare baselines characteristics. Since the triglyceride level did not show a normal distribution, the Kruskal–Wallis test was used to compare the triglyceride level between groups. Analysis of covariance (ANCOVA) was used to compare the RBANS index scores among the groups, adjusted for baseline score. The independent *t*-test was used for the frequency band power of each channel on the 68 ROIs and iCoh among the 68 ROIs between the intervention and control groups. Since changes in characteristic path length of each frequency band in the 68 ROIs did not show a normal distribution, Mann-Whitney U test was used to compare changes in the characteristic path length of each frequency band between groups. And to deal with missing data, multiple imputations were performed using a fully conditional specification implemented as a MICE algorithm (van Buuren and Groothuis-Oudshoorn, 2011). We performed predictive mean matching with 20 iterations of the imputation model. For this analysis, the MICE package of R statistical software version 4.0.5 (R Foundation^2^) was used. Linear regression adjusted for age, sex, and education was used to examine the relationship between the change in the characteristic path length of each frequency band in each of the 68 ROIs and the change in the RBANS index score in each of the FMI and HMI groups. The significance of each *p* value in the 68 ROIs was tested by controlling the false discovery rate (FDR) with the Benjamini-Hochberg procedure for multiple testing corrections (Benjamini and Hochberg, 1995). Statistical analyses were performed with IBM SPSS version 26 (IBM, Armonk, NY, USA). Statistical significance was set at *p* < 0.05.

## Results

The baseline clinical characteristics of all participants are shown in Table 1. No differences were found in demographic factors, medical history, vascular risk factors, lifestyle factors, and cognition among the three groups. Changes in the total scale index score (*p* = 0.002) and visuoconstruction index scores (*p* < 0.001) of the RBANS between pre-intervention and post-intervention showed improvement in all intervention groups including FMI and HMI groups, compared to the control group. Compared with the control group, the RBANS total scale index score and the visuoconstruction index score were also significantly improved in each of the FMI and HMI groups (Table 2). In addition, a statistical trend toward improvement was observed in the attention index score in the HMI group (*p* = 0.099) and in the delayed memory index score in the FMI group (*p* = 0.050).

**TABLE 1:** Clinical characteristics of all participants in this study.

	FMI group (*N* = 44)	HMI group (*N* = 49)	Control group (*N* = 34)	*P-*value
**Demographic factors**				
Age at baseline, years	71.5 ± 5.0	70.9 ± 5.0	69.6 ± 4.9	0.225
Number of women	31 (70.56%)	36 (73.5%)	28 (82.4%)	0.485
Education, years	10.1 ± 4.7	9.9 ± 4.9	10.1 ± 4.9	0.976
Obesity	14 (31.8%)	23 (46.9%)	13 (40.6%)	0.318
Abdominal obesity	15 (34.1%)	20 (40.8%)	16 (47.1%)	0.507
Low physical activity	21 (47.7%)	26 (53.1%)	16 (47.1%)	0.825
Low social activity	10 (22.7%)	13 (26.5%)	11 (34.4%)	0.525
Apolipoprotein E ε4 carrier	6 (13.6%)	13 (26.5%)	5 (14.7%)	0.247
Family history of dementia	7 (15.9%)	14 (28.6%)	6 (17.6%)	0.290
**Medical history**				
Hypertension	21 (47.7%)	26 (53.1%)	17 (50.0%)	0.878
Diabetes mellitus	8 (18.2%)	13 (26.5%)	9 (26.5%)	0.576
Dyslipidemia	20 (45.5%)	26 (53.1%)	20 (58.8%)	0.493
Cardiac disease	4 (9.1%)	3 (6.1%)	2 (5.9%)	0.829
History of stroke	4 (9.1%)	3 (6.1%)	6 (17.6%)	0.230
Mild cognitive impairment	15 (34.1%)	15 (30.6%)	5 (14.7%)	0.144
**Vascular factors**				
Systolic blood pressure, mmHg	128.3 ± 16.1	127.1 ± 12.9	131.7 ± 17.3	0.402
Diastolic blood pressure, mmHg	74.5 ± 10.5	74.4 ± 10.3	74.8 ± 8.4	0.987
Total cholesterol, mg/dl	185.6 ± 38.2	189.2 ± 36.2	176.2 ± 42.2	0.322
LDL-cholesterol, mg/dl	105.1 ± 36.1	109.9 ± 33.5	98.5 ± 32.2	0.329
Triglyceride, mg/dl^†^	117.0 (78.0, 175.0)	113.0 (91.0, 194.0)	112.0 (82.0, 147.5)	0.875
HDL-cholesterol, mg/dl	53.8 ± 12.7	53.9 ± 14.6	52.9 ± 14.2	0.938
Body Mass Index, kg/m^2^	23.7 ± 2.1	24.2 ± 3.1	25.3 ± 3.0	0.056
Abdominal circumference, cm	82.8 ± 7.2	84.5 ± 9.4	85.9 ± 8.4	0.256
**Lifestyle factors**				
Current smokers	2 (4.5%)	1 (2.0%)	0 (0.0%)	0.625
Current alcohol drinkers	12 (27.3%)	8 (16.3%)	10 (29.4%)	0.294
**Cognition**				
K-MMSE	28.2 ± 1.7	27.9 ± 1.8	27.3 ± 2.5	0.123
RBANS total scale index score	101.4 ± 19.5	101.0 ± 16.1	103.6 ± 18.2	0.795

Values are shown as the mean ± SD or *n* (%). FMI, facility-based multidomain intervention; HMI, home-based multidomain intervention; LDL, low density lipoprotein; HDL, high density lipoprotein; K-MMSE, Korean Mini-Mental State Examination; RBANS, Repeatable Battery for the Assessment of Neuropsychological Status. *P*-value was calculated from the one-way of variance for the numerical data or the chi-square test for the categorical data.

^†^Results are median and interquartile range and *p*-value was calculated from the Kruskal–Wallis test.

**TABLE 2:** Mean changes in the index scores of the Repeatable Battery for the Assessment of Neuropsychological Status.

Index score	Baseline scores				Changes from baseline to study end				*P*-value*		
	FMI (*n* = 44)	HMI (*n* = 49)	FMI + HMI (*n* = 93)	Control (*n* = 34)	FMI (*n* = 44)	HMI (*n* = 49)	FMI + HMI (*n* = 93)	Control (*n* = 34)	FMI vs. control	HMI vs. control	FMI + HMI vs. control
Total scale	101.4 ± 19.5	101.0 ± 16.1	101.2 ± 17.7	103.6 ± 18.2	5.4 ± 7.7	5.4 ± 8.2	5.4 ± 7.9	–0.03 ± 9.3	0.006	0.007	0.002
Immediate memory	102.2 ± 13.6	99.0 ± 14.7	100.5 ± 14.2	102.2 ± 14.5	4.7 ± 9.3	3.5 ± 12.7	4.1 ± 11.2	3.6 ± 10.3	0.603	0.655	0.995
Visuoconstruction	93.6 ± 16.8	94.2 ± 15.5	93.9 ± 16.0	93.7 ± 13.9	1.2 ± 14.2	0.8 ± 14.6	1.0 ± 14.3	–8.5 ± 11.3	0.001	0.001	<0.001
Language	104.9 ± 15.9	108.2 ± 13.4	106.6 ± 14.7	108.7 ± 12.9	1.4 ± 12.5	1.7 ± 12.6	1.6 ± 12.5	0.6 ± 11.2	0.539	0.686	0.933
Attention	104.4 ± 18.5	102.8 ± 16.6	103.6 ± 17.5	101.8 ± 17.5	0.02 ± 8.5	2.5 ± 9.6	1.3 ± 9.1	–0.5 ± 10.8	0.588	0.099	0.208
Delayed memory	94.5 ± 16.5	94.1 ± 14.6	94.3 ± 15.5	100.4 ± 16.4	10.3 ± 10.2	9.2 ± 12.1	9.7 ± 11.2	4.6 ± 9.3	0.050	0.383	0.139

Values are shown as the mean ± SD. RBANS, Repeatable Battery for the Assessment of Neuropsychological Status; FMI, facility-based multidomain intervention; HMI, home-based multidomain intervention.

*Analysis of covariance with each baseline score as a covariate.

### Changes in quantitative electroencephalography parameters in all intervention groups

**FIGURE 2:**
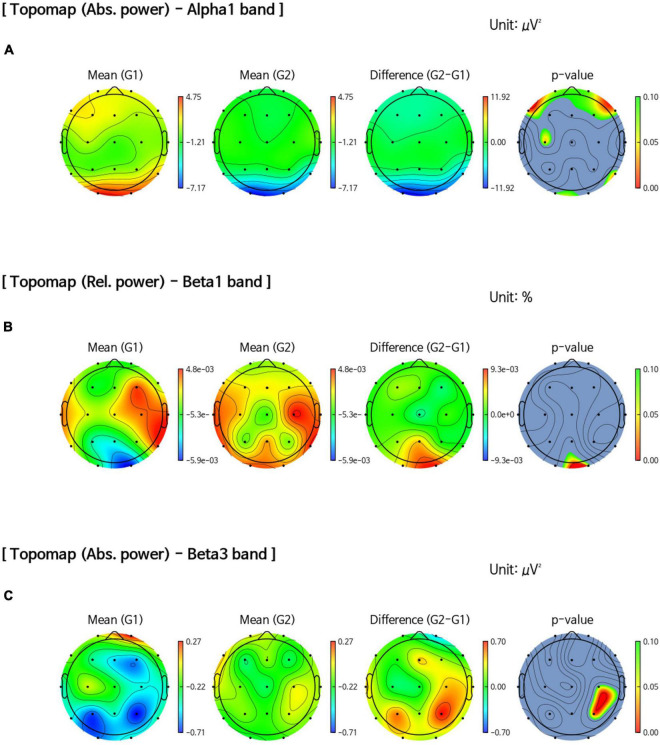
Comparison of band power changes between all intervention and control groups. **(A)** Difference between post-intervention and pre-intervention in the control group (G1) showed an increase in the absolute power of the alpha1 band in the frontal, central, and temporal regions than in all intervention groups (G2). **(B)** An increase in the relative power of the beta1 band in the occipital region (*p* = 0.041) after the intervention was observed in all intervention groups (G2) compared with the control group (G1). **(C)** An increase in the absolute power of the beta3 band in the right parietal region (*p* = 0.022) after the intervention was observed in all intervention groups (G2) compared with the control group (G1).

**FIGURE 3:**
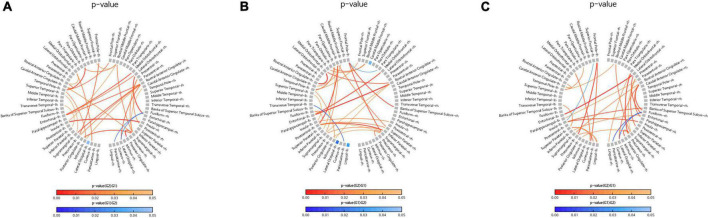
Comparison of changes in the imaginary part of coherence (iCoh) of the alpha1 band. Regions with significant differences in the iCoh changes between two groups are shown in the figures. Red lines represent a significant increase in the iCoh of the alpha1 band in the intervention group (G2) than in the control group (G1). Blue lines represent a significant increase in the iCoh of the alpha1 band in the control group (G1) than in the intervention group (G2). **(A)** Comparison of the control group (G1) with all intervention groups (G2), including facility-based multidomain intervention (FMI) and home-based multidomain intervention (HMI) groups. **(B)** Comparison of the FMI group (G2) with the control group (G1). **(C)** Comparison of the HMI group (G2) with the control group (G1).

The sensor-level analysis of EEG showed that an accelerating pattern of rhythm with alph1 decreased at F7 (*p* = 0.026), F8 (*p* = 0.044), C3 (*p* = 0.049), and T6 (*p* = 0.040) in all intervention groups, including the FMI and HMI groups, compared with the control group (Figure 2A). In addition, increases in the relative power of the beta1 band in the occipital region (*p* = 0.041) and in the absolute power of the beta3 band in the right parietal region (*p* = 0.022) were observed in all intervention groups compared with the control group (Figures 2B,C). Although these differences were not statistical significant (*p* = 0.079), the intervention groups showed an increasing tendency of occipital alpha peak frequency (mean difference = 0.017) in the O2 area, whereas the control group showed the opposite pattern (mean difference = −0.242). The functional connectivity analysis showed an increase in the iCoh of the alpha1 band, the default resting-state oscillating rhythm, in all intervention groups, whereas the control group showed the opposite results (Figure 3A). Compared to the control group, the characteristic path length of alpha1 band was decreased in the right supramarginal gyrus (*p* = 0.003) and right rostral middle frontal cortex (*p* = 0.003) in all intervention groups after multiple imputation for missing data (Table 3).

**TABLE 3:** Comparison of changes in characteristic path length of alpha1 band between the intervention and control groups.

	Changes from baseline to study end excluding missing data				Changes from baseline to study end after multiple imputation for missing data			
	FMI (*n* = 44)	HMI (*n* = 49)	FMI + HMI (*n* = 93)	Control (*n* = 34)	FMI (*n* = 51)	HMI (*n* = 51)	FMI + HMI (*n* = 102)	Control (*n* = 50)
Rt. Supramarginal gyrus	−0.04 (−0.17 ∼ 0.20) *p* = 0.030	−0.03 (−0.26 ∼ 0.14) *p* = 0.009*	−0.03 (−0.23 ∼ 0.15) *p* = 0.007*	0.09 (0.01 ∼ 0.25)	−0.03 (−0.17 ∼ 0.23) *p* = 0.032	−0.03 (−0.27 ∼ 0.13) *p* = 0.002*	−0.03 (−0.23 ∼ 0.16) *p* = 0.003*	0.10 (−0.03 ∼ 0.30)
Rt. Rostral middle frontal cortex	−0.03 (−0.33 ∼ 0.19) *p* = 0.020	0.00 (−0.37 ∼ 0.25) *p* = 0.042	−0.02 (−0.33 ∼ 0.22) *p* = 0.014	0.16 (−0.03 ∼ 0.44)	−0.05 (−0.27 ∼ 0.19) *p* = 0.007*	0.00 (−0.42 ∼ 0.29) *p* = 0.014	−0.03 (−0.32 ∼ 0.22) *p* = 0.003*	0.16 (−0.13 ∼ 1.07)
Rt. Lateral occipital cortex	0.10 (−0.10 ∼ 0.34) *p* = 0.001*	0.14 (−0.16 ∼ 0.32) *p* = 0.001*	0.12 (−0.11 ∼ 0.33) *p* < 0.001*	−0.10 (−0.44 ∼ 0.06)	0.06 (−0.11 ∼ 0.33) *p* = 0.002*	0.14 (−0.14 ∼ 0.33) *p* < 0.001*	0.11 (−0.12 ∼ 0.33) *p* < 0.001*	−0.10 (−0.44 ∼ 0.10)
Rt. Cuneus	0.05 (−0.16 ∼ 0.39) *p* = 0.052	0.05 (−0.15 ∼ 0.28) *p* = 0.057	0.05 (−0.15 ∼ 0.31) *p* = 0.030	−0.03 (−0.32 ∼ 0.18)	0.02 (−0.17 ∼ 0.39) *p* = 0.029	0.03 (−0.16 ∼ 0.28) *p* = 0.048	0.02 (−0.16 ∼ 0.31) *p* = 0.016	−0.03 (−0.34 ∼ 0.18)
Lt. Temporal pole	0.01 (−0.17 ∼ 0.16) *p* = 0.208	−0.09 (−0.25 ∼ 0.07) *p* = 0.007*	−0.06 (−0.21 ∼ 0.12) *p* = 0.023	0.09 (−0.10 ∼ 0.22)	0.03 (−0.17 ∼ 0.19) *p* = 0.441	−0.09 (−0.25 ∼ 0.09) *p* = 0.012	−0.05 (−0.21 ∼ 0.14) *p* = 0.057	0.09 (−0.13 ∼ 0.22)

Values are shown as median (IQL). *P*-values represent comparisons with the control group by Mann–Whitney *U* test.

*Statistically significant after controlling the false discovery rate. FMI, facility-based multidomain intervention; HMI, home-based multidomain intervention; Rt., right; Lt., left.

### Changes in quantitative electroencephalography parameters in facility-based multidomain intervention group

In the FMI group compared to the control group, sensor-level analysis showed a decrease in the absolute power of the alpha2 band (*p* = 0.034) in the temporal area and a decrease in the relative power of the alpha1 band (*p* = 0.035) in the left temporal cortex (T3). Additionally, an increase in the absolute power of the beta3 band was shown in the right parietal area (P4, *p* = 0.038) in the FMI group compared to the control group. FMI group exhibited an increasing tendency of the occipital alpha peak frequency in the O2 area (mean difference = 0.069) compared to the controls (mean difference = −0.242), but the difference was not statistically significant (*p* = 0.089). The functional connectivity analysis showed an increase in the iCoh of the alpha1 band, the default resting-state oscillating rhythm, in the FMI group compared to the control group (Figure 3B). Brain network analysis showed a decreased characteristic path length of alpha1 band in the right rostral middle frontal cortex (*p* = 0.007) in the FMI group compared to the control group (Table 3). The control group showed a decrease in characteristic path length (*p* = 0.002) compared with the FMI group in the right lateral occipital cortex (Table 3).

### Changes in quantitative electroencephalography parameters in home-based multidomain intervention group

In a comparison between the HMI and the control groups, the HMI group showed an accelerating alpha1 brain rhythm pattern in the frontal (F7, *p* = 0.027; F8, *p* = 0.048), central (C3, *p* = 0.047), and temporal regions (T6, *p* = 0.036) decreased more than in the controls whereas the relative beta1 band in the frontal region (F3, *p* = 0.047) increased more in the HMI than in the control group. The functional connectivity analysis showed an increase in the iCoh of the alpha1 band in the HMI group compared to the control group (Figure 3C). Brain network analysis revealed that the characteristic path length of the alpha1 band was decreased in the right supramarginal gyrus (*p* = 0.009) and left temporal pole area (*p* = 0.007) in the HMI group compared with the control group (Table 3).

### Associations between characteristic path length change and Repeatable Battery for the Assessment of Neuropsychological Status change

The improvement in the RBANS total scale index score was associated with a decrease in the characteristic path length of alpha1 band in the left medial orbitofrontal cortex in the FMI group and in the right posterior central cortex in the HMI group (Table 4). The improvement in the visuoconstruction index score of the RBANS was associated with a decrease in the characteristic path length of the alpha1 band in the left parahippocampal cortex and right frontal pole in the FMI group. There was no association between the change in the characteristic path length of the alpha1 band in each of the 68 ROIs and the change in the index score of other cognitive domains of the RBANS in the FMI group. There was no association between the change in the characteristic path length of the alpha1 band in each of the 68 ROIs and the change in the index score of all five cognitive domains of the RBANS in the HMI group.

**TABLE 4:** Associations between changes in the characteristic path length of alpha1 band and changes in the Repeatable Battery for the Assessment of Neuropsychological Status in each intervention group.

	FMI group		HMI group
	RBANS total scale index score	Visuoconstruction index score	RBANS total scale index score
Lt. Medial orbitofrontal cortex	–0.057 (–0.086 ∼ -0.029) *p* < 0.001*	–0.022 (–0.040 ∼ -0.003) *p* = 0.022	0.003 (–0.017 ∼ 0.024) *p* = 0.753
Lt. Inferior parietal cortex	–0.009 (–0.043 ∼ 0.025) *p* = 0.593	–0.008 (–0.027 ∼ 0.011) *p* = 0.407	0.027 (0.008 ∼ 0.045) *p* = 0.005*
Lt. parahippocampal cortex	–0.010 (–0.022 ∼ 0.002) *p* = 0.106	–0.010 (–0.016 ∼ -0.003) *p* = 0.004*	0.007 (–0.003 ∼ 0.016) *p* = 0.175
Rt. Frontal pole	–0.032 (–0.077 ∼ 0.014) *p* = 0.171	–0.035 (–0.060 ∼ -0.010) *p* = 0.007*	0.020 (–0.026 ∼ 0.066) *p* = 0.377
Rt. Posterior central cortex	0.001 (–0.023 ∼ 0.026) *p* = 0.919	0.003 (–0.011 ∼ 0.017) *p* = 0.653	–0.019 (–0.035 ∼ -0.003) *p* = 0.002*

Values are shown as b (95% CI). *P*-value was calculated by linear regression analysis adjusted for age, sex, and education.

*Statistically significant after controlling the false discovery rate. RBANS, Repeatable Battery for the Assessment of Neuropsychological Status; FMI, facility-based multidomain intervention; HMI, home-based multidomain intervention; Rt., right; Lt., left.

## Discussion

This is the first study that has used QEEG to investigate functional brain changes following a multidomain lifestyle intervention program to prevent dementia. This study found that the intervention group exhibited increases in the iCoh of the alpha1 band and in the relative power of the beta1 band and the absolute power of the beta3 band as well as a decrease in the characteristic path length of alpha1 band compared to the controls. Additionally, a negative association between changes of the RBANS total scale index score and changes in the characteristic path length of the alpha1 band was shown in the FMI and HMI groups, respectively.

The increased iCoh of the alpha1 band in the intervention group may be an important biological marker for improved cognition after a 24-week multidomain lifestyle intervention program. Coherence is a measure of the degree of synchronization among EEG signals from different brain regions (Hogan et al., 2003), and the iCoh has been interpreted as a measure of brain connectivity (Nolte et al., 2004). Increased iCoh of the alpha1 band in this study implied increased connectivity of the alpha1 band, in contrast to earlier findings showing a reduction in alpha coherence in AD patients (Locatelli et al., 1998; Adler et al., 2003; Hogan et al., 2003). AD is a cortical disconnection syndrome, which refers to disruptions of structural and functional connectivity in topographically dispersed brain regions (Brier et al., 2014). In addition, the cholinergic system plays a role in the modulation of intracortical connectivity; therefore, it is not surprising that functional connectivity is disrupted in AD patients with a cholinergic deficit. Furthermore, interhemispheric coherence decreases with advanced age in normal older adults (Duffy et al., 1996; Kikuchi et al., 2000). In this regard, the increased iCoh among at-risk older adults in the intervention group suggested a positive change in functional connectivity and might be associated with improvement in the RBANS total scale index score in the intervention group. The intervention group showed a 5-point increase in the total scale index score of the RBANS and an increase in the iCoh of the alpha1 band. In contrast, the control group showed no change in the same index score and exhibited a decrease in the iCoh similar to that in normal older adults. Therefore, an increase in the iCoh of the alpha1 band may be the earliest change and may serve as a neurophysiological marker, providing evidence for biological effects on cognition in response to a multidomain lifestyle intervention.

The increase in the relative beta1 power and absolute beta3 power may be an electrophysiological marker for improvement in cognition in this study. In AD patients, a decrease in alpha power and beta power as well as an increase in theta power and delta power were shown previously (Garn et al., 2014). Furthermore, relative parietal beta1 power showed a negative correlation with amyloid deposition and a positive correlation with anterograde memory in MCI patients (Musaeus et al., 2018). Decreased relative power in the beta1 band could be a predictive marker for progression in MCI patients (Musaeus et al., 2018). In contrast to previous findings in AD and MCI patients, the present study found increased relative power in the beta1 band in the intervention group, which also showed improvement in the visuoconstruction index score on the RBANS. An increase in beta band power reflects top-down attentional modulation between brain areas by promoting feedback interactions across visual areas (Bastos et al., 2015). In addition, alterations in the beta band are associated with the resting-state EEG default mode network (DMN) (Chen et al., 2008). In this regard, increased power in the beta1 and beta3 bands might suggest functional restoration of the EEG DMN, specifically top-down attentional process.

Also of note is the decrease in the characteristic path length found in the intervention group. Quantitative analysis of complex brain networks is based on graph theory, with brain networks defined as a set of nodes or vertices and the edges or lines between them. When investigating altered features of functional brain networks, EEG provides measurement of neuronal activity with good temporal resolution (Bullmore and Sporns, 2009). AD is characterized by a loss of small-world network characteristics, as seen in the longer characteristic path length (Stam et al., 2007). As path length is defined by the minimum number of edges, a longer path length represents lower global efficiency. An increase in path length was also shown in MCI patients, and it was negatively correlated with cognitive status (Zeng et al., 2015). In this study, we found a decrease in characteristic path length in the intervention group, but an increased path length in the control group (Table 3). This finding implied a positive shift toward the restoration of functional brain networks through the multidomain intervention program, in contrast to a loss of small-world network characteristics in MCI or AD patients from the perspective of a disconnection syndrome.

Finally, results showed a negative association between improvement in cognitive status and a decrease in the characteristic path length of the alpha1 band. Increased global efficiency, measured by the characteristic path length, was associated with an increase in the RBANS total scale index score in each of the FMI and HMI groups. This finding is in line with previous reports showing correlations between EEG parameters and neuropsychological test scores despite of various EEG parameters (Brunovsky et al., 2003; Babiloni et al., 2006). Specifically, the negative correlation between a cognitive marker and a marker of global efficiency in this study contrasts with previous findings showing a positive correlation between small-worldness and Montreal Cognitive Assessment scores in MCI and AD patients (Frantzidis et al., 2014; Zeng et al., 2015), suggesting the functional restoration of network organization as cognition improves.

This study has some limitations. First, a 24-week period for a multidomain lifestyle intervention program may be too short to confirm changes in EEG parameters. This study did not find an increase in the alpha band or a decrease in the delta or theta band. However, despite the intervention program’s short duration, an increase in the iCoh of the alpha1 band and a decrease in the characteristic path length of the alpha1 band in the intervention group suggested very early changes in neurophysiological markers following a lifestyle modification program. Second, this study was originally planned as a feasibility study with a small sample size in contrast to previous multidomain intervention studies with large sample sizes. The small sample size may underestimate the positive results of this study when we assess the biological effects of a multidomain intervention study to prevent dementia. However, the sample size in this study was comparable to that of previous studies of single or combined intervention programs using EEG. Third, this study did not assess biomarkers of AD including amyloid and tau, so information on the subjects’ pathologic status was not available. The biological changes in EEG parameters after the intervention program might vary according to pathologic status. For example, subjects with preclinical AD might be less likely to show functional changes after an intervention program than would normal older adults without amyloid deposition. Fourth, there were more participants in the control group for whom follow-up EEG was not performed than in the intervention group. It is possible that this may have influenced the study results. However, since the participants excluded from the EEG analysis in the control group were older, it is likely that it may have a more favorable effect on the results of the control group, and it is not likely that it affected the false positives of the results of this study. Additionally, the results of comparing changes in the characteristic path length of the alpha1 band between the intervention and control groups after excluding missing data were similar to those in the analysis after multiple imputation for missing data.

In summary, this study is the first study to show positive functional brain changes using QEEG after a 24-week multidomain lifestyle intervention to prevent dementia. The increased iCoh and the decreased characteristic path length of the alpha1 band in the intervention group implied increased functional brain networks with higher global efficiency following a lifestyle intervention program in at-risk older adults. Further studies with larger sample sizes and/or a longer period of intervention are needed to confirm the findings of this study.

## Data availability statement

Anonymized data used in this work will be available from the corresponding authors upon request.

## Ethics statement

The studies involving human participants were reviewed and approved by Inha University Hospital Institutional Review Board (IRB)(INHAUH-2018-11-022), Ewha Womans University Mokdong Hospital IRB (EUMC-2019-04-013), Ajou University Hospital IRB (AJIRB-BMR-SUR-19-070 and AJIRB-BMR-SUR-19-077), Dong-A University Hospital IRB (DAUHIRB-19-078), Chonnam National University IRB (CNUH-2019-139). The patients/participants provided their written informed consent to participate in this study.

## Author contributions

HKP, SHChoi, SK, SWK, HRN, and YKP contributed to the study concept and design. HKP, SHChoi, JHJ, SYM, CHH, MC, H-SS, B-OC, SML, KWP, BCK, SHCho, HRN, and YKP participated in data collection and processing. HKP, SHChoi, SK, and UP performed the statistical analysis. HKP, SHChoi, SK, SWK, HRN, and YKP contributed to the analysis and interpretation of data and wrote the manuscript. All authors participated in the critical revision of the manuscript and final approval of the version.

## Footnotes

1
https://isyncbrain.com/

2
https://www.r-project.org

## References

[B1] Adler G. Brassen S. Chwalek K. Dieter B. Teufel M. (2004). Prediction of treatment response to rivastigmine in Alzheimer’s dementia. *J. Neurol. Neurosurg. Psychiatry* 75 292–294. 14742608 PMC1738912

[B2] Adler G. Brassen S. Jajcevic A. (2003). EEG coherence in Alzheimer’s dementia. *J. Neural Transm.* 110 1051–1058. 10.1007/s00702-003-0024-8 12928837

[B3] Andrieu S. Guyonnet S. Coley N. Cantet C. Bonnefoy M. Bordes S. (2017). Effect of long-term omega 3 polyunsaturated fatty acid supplementation with or without multidomain intervention on cognitive function in elderly adults with memory complaints (MAPT): A randomised, placebo-controlled trial. *Lancet Neurol.* 16 377–389. 10.1016/S1474-4422(17)30040-6 28359749

[B4] Babiloni C. Carducci F. Lizio R. Vecchio F. Baglieri A. Bernardini S. (2013). Resting state cortical electroencephalographic rhythms are related to gray matter volume in subjects with mild cognitive impairment and Alzheimer’s disease. *Hum. Brain Mapp.* 34 1427–1446. 10.1002/hbm.22005 22331654 PMC6869852

[B5] Babiloni C. Del Percio C. Boccardi M. Lizio R. Lopez S. Carducci F. (2015). Occipital sources of resting-state alpha rhythms are related to local gray matter density in subjects with amnesic mild cognitive impairment and Alzheimer’s disease. *Neurobiol. Aging* 36 556–570. 10.1016/j.neurobiolaging.2014.09.011 25442118 PMC4315728

[B6] Babiloni C. Frisoni G. Steriade M. Bresciani L. Binetti G. Del Percio C. (2006). Frontal white matter volume and delta EEG sources negatively correlate in awake subjects with mild cognitive impairment and Alzheimer’s disease. *Clin. Neurophysiol.* 117 1113–1129. 10.1016/j.clinph.2006.01.020 16564740

[B7] Babiloni C. Frisoni G. B. Pievani M. Vecchio F. Lizio R. Buttiglione M. (2009). Hippocampal volume and cortical sources of EEG alpha rhythms in mild cognitive impairment and Alzheimer disease. *Neuroimage* 44 123–135. 10.1016/j.neuroimage.2008.08.005 18805495

[B8] Bastos A. M. Vezoli J. Bosman C. A. Schoffelen J. Oostenveld R. Dowdall J. R. (2015). Visual areas exert feedforward and feedback influences through distinct frequency channels. *Neuron* 85 390–401. 10.1016/j.neuron.2014.12.018 25556836

[B9] Benjamini Y. Hochberg Y. (1995). Controlling the false discovery rate: A practical and powerful approach to multiple testing. *J. R. Stat. Soc. Ser. B Methodol.* 57 289–300. 10.1111/j.2517-6161.1995.tb02031.x

[B10] Brier M. R. Thomas J. B. Ances B. M. (2014). Network dysfunction in Alzheimer’s disease: Refining the disconnection hypothesis. *Brain Connect.* 4 299–311. 10.1089/brain.2014.0236 24796856 PMC4064730

[B11] Brunovsky M. Matousek M. Edman A. Cervena K. Krajca V. (2003). Objective assessment of the degree of dementia by means of EEG. *Neuropsychobiology* 48 19–26. 10.1159/000071824 12886036

[B12] Bullmore E. Sporns O. (2009). Complex brain networks: Graph theoretical analysis of structural and functional systems. *Nat. Rev. Neurosci.* 10 186–198. 19190637 10.1038/nrn2575

[B13] Chen A. C. Feng W. Zhao H. Yin Y. Wang P. (2008). EEG default mode network in the human brain: Spectral regional field powers. *Neuroimage* 41 561–574. 10.1016/j.neuroimage.2007.12.064 18403217

[B14] Chin J. Park J. Yang S. J. Yeom J. Ahn Y. Baek M. J. (2018). Re-standardization of the Korean-instrumental activities of daily living (K-IADL): Clinical usefulness for various neurodegenerative diseases. *Dement. Neurocogn. Disord.* 17 11–22. 10.12779/dnd.2018.17.1.11 30906387 PMC6427997

[B15] Duffy F. H. Mcanulty G. B. Albert M. S. (1996). Effects of age upon interhemispheric EEG coherence in normal adults. *Neurobiol. Aging* 17 587–599. 10.1016/0197-4580(96)00007-3 8832634

[B16] Engels M. Stam C. J. van der Flier W. M. Scheltens P. de Waal H. (2015). Declining functional connectivity and changing hub locations in Alzheimer’s disease: An EEG study. *BMC Neurol.* 15:145. 10.1186/s12883-015-0400-7 26289045 PMC4545875

[B17] Frantzidis C. A. Vivas A. B. Tsolaki A. Klados M. A. Tsolaki M. Bamidis P. D. (2014). Functional disorganization of small-world brain networks in mild Alzheimer’s disease and amnestic mild cognitive impairment: An EEG study using relative wavelet entropy (RWE). *Front. Aging Neurosci.* 6:224. 10.3389/fnagi.2014.00224 25206333 PMC4144118

[B18] Gandelman-Marton R. Aichenbaum S. Dobronevsky E. Khaigrekht M. Rabey J. M. (2017). Quantitative EEG after brain stimulation and cognitive training in Alzheimer disease. *J. Clin. Neurophysiol.* 34 49–54. 10.1097/WNP.0000000000000301 28045857

[B19] Garn H. Waser M. Deistler M. Schmidt R. Dal-Bianco P. Ransmayr G. (2014). Quantitative EEG in Alzheimer’s disease: Cognitive state, resting state and association with disease severity. *Int. J. Psychophysiol.* 93 390–397. 10.1016/j.ijpsycho.2014.06.003 24933410

[B20] Gianotti L. R. Künig G. Faber P. L. Lehmann D. Pascual-Marqui R. D. Kochi K. (2008). Rivastigmine effects on EEG spectra and three-dimensional LORETA functional imaging in Alzheimer’s disease. *Psychopharmacology* 198 323–332. 10.1007/s00213-008-1111-1 18446328

[B21] Han S. Pyun J. Yeo S. Kang D. W. Jeong H. T. Kang S. W. (2021). Differences between memory encoding and retrieval failure in mild cognitive impairment: Results from quantitative electroencephalography and magnetic resonance volumetry. *Alzheimers Res. Ther.* 13:3. 10.1186/s13195-020-00739-7 33397486 PMC7784298

[B22] Hassan M. Dufor O. Merlet I. Berrou C. Wendling F. (2014). EEG source connectivity analysis: From dense array recordings to brain networks. *PLoS One* 9:e105041. 10.1371/journal.pone.0105041 25115932 PMC4130623

[B23] Hogan M. J. Swanwick G. R. Kaiser J. Rowan M. Lawlor B. (2003). Memory-related EEG power and coherence reductions in mild Alzheimer’s disease. *Int. J. Psychophysiol.* 49 147–163. 10.1016/s0167-8760(03)00118-1 12919717

[B24] Huang P. Fang R. Li B. Chen S. (2016). Exercise-related changes of networks in aging and mild cognitive impairment brain. *Front. Aging Neurosci.* 8:47. 10.3389/fnagi.2016.00047 27014055 PMC4779936

[B25] Jelic V. Blomberg M. Dierks T. Basun H. Shigeta M. Julin P. (1998). EEG slowing and cerebrospinal fluid tau levels in patients with cognitive decline. *Neuroreport* 9 157–160. 9592068 10.1097/00001756-199801050-00032

[B26] Jeong J. (2004). EEG dynamics in patients with Alzheimer’s disease. *Clin. Neurophysiol.* 115 1490–1505. 15203050 10.1016/j.clinph.2004.01.001

[B27] Kikuchi M. Wada Y. Koshino Y. Nanbu Y. Hashimoto T. (2000). Effect of normal aging upon interhemispheric EEG coherence: Analysis during rest and photic stimulation. *Clin. Electroencephalogr.* 31 170–174. 10.1177/155005940003100404 11056838

[B28] Kogan E. A. Korczyn A. D. Virchovsky R. G. Klimovizky S. S. Treves T. A. Neufeld M. Y. (2001). EEG changes during long-term treatment with donepezil in Alzheimer’s disease patients. *J. Neural Transm.* 108 1167–1173. 10.1007/s007020170006 11725819

[B29] Lejko N. Larabi D. I. Herrmann C. S. Aleman A. Ćurčić-Blake B. (2020). Alpha power and functional connectivity in cognitive decline: A systematic review and meta-analysis. *J. Alzheimers Dis.* 78 1047–1088. 10.3233/JAD-200962 33185607 PMC7739973

[B30] Liu J. Li M. Pan Y. Lan W. Zheng R. Wu F. (2017). Complex brain network analysis and its applications to brain disorders: A survey. *Complexity* 2017:8362741. 10.1155/2017/8362741

[B31] Locatelli T. Cursi M. Liberati D. Franceschi M. Comi G. (1998). EEG coherence in Alzheimer’s disease. *Electroencephalogr. Clin. Neurophysiol.* 106 229–237. 10.1016/S0013-4694(97)00129-6 9743281

[B32] Moon S. Y. Hong C. H. Jeong J. H. Park Y. K. Na H. R. Song H. (2021). Facility-based and home-based multidomain interventions including cognitive training, exercise, diet, vascular risk management, and motivation for older adults: A randomized controlled feasibility trial. *Aging* 13:15898. 10.18632/aging.203213 34148030 PMC8266338

[B33] Musaeus C. S. Nielsen M. S. Østerbye N. N. Høgh P. (2018). Decreased parietal beta power as a sign of disease progression in patients with mild cognitive impairment. *J. Alzheimers Dis.* 65 475–487. 10.3233/JAD-180384 30056426

[B34] Ngandu T. Lehtisalo J. Solomon A. Levälahti E. Ahtiluoto S. Antikainen R. (2015). A 2 year multidomain intervention of diet, exercise, cognitive training, and vascular risk monitoring versus control to prevent cognitive decline in at-risk elderly people (FINGER): A randomised controlled trial. *Lancet* 385 2255–2263. 10.1016/S0140-6736(15)60461-5 25771249

[B35] Nolte G. Bai O. Wheaton L. Mari Z. Vorbach S. Hallett M. (2004). Identifying true brain interaction from EEG data using the imaginary part of coherency. *Clin. Neurophysiol.* 115 2292–2307. 10.1016/j.clinph.2004.04.029 15351371

[B36] Park H. K. Jeong J. H. Moon S. Y. Park Y. K. Hong C. H. Na H. R. (2020). South Korean study to prevent cognitive impairment and protect brain health through lifestyle intervention in at-risk elderly people: Protocol of a multicenter, randomized controlled feasibility trial. *J. Clin. Neurol.* 16 292–303. 10.3988/jcn.2020.16.2.292 32319247 PMC7174118

[B37] Roh J. H. Park M. H. Ko D. Park K. Lee D. Han C. (2011). Region and frequency specific changes of spectral power in Alzheimer’s disease and mild cognitive impairment. *Clin. Neurophysiol.* 122 2169–2176. 10.1016/j.clinph.2011.03.023 21715226

[B38] Rubinov M. Sporns O. (2010). Complex network measures of brain connectivity: Uses and interpretations. *Neuroimage* 52 1059–1069. 10.1016/j.neuroimage.2009.10.003 19819337

[B39] Smailovic U. Jelic V. (2019). Neurophysiological markers of Alzheimer’s disease: Quantitative EEG approach. *Neurol. Ther.* 8 37–55. 10.1007/s40120-019-00169-0 31833023 PMC6908537

[B40] Smailovic U. Koenig T. Kåreholt I. Andersson T. Kramberger M. G. Winblad B. (2018). Quantitative EEG power and synchronization correlate with Alzheimer’s disease CSF biomarkers. *Neurobiol. Aging* 63 88–95. 10.1016/j.neurobiolaging.2017.11.005 29245058

[B41] Stam C. J. Jones B. F. Nolte G. Breakspear M. Scheltens P. (2007). Small-world networks and functional connectivity in Alzheimer’s disease. *Cereb. Cortex* 17 92–99. 10.1093/cercor/bhj127 16452642

[B42] Stephen R. Liu Y. Ngandu T. Antikainen R. Hulkkonen J. Koikkalainen J. (2019). Brain volumes and cortical thickness on MRI in the Finnish geriatric intervention study to prevent cognitive impairment and disability (FINGER). *Alzheimers Res. Ther.* 11:53. 31164160 10.1186/s13195-019-0506-z PMC6549301

[B43] van Buuren S. Groothuis-Oudshoorn K. (2011). mice: Multivariate imputation by chained equations in R. *J. Stat. Softw.* 45 1–67.

[B44] Xia M. Wang J. He Y. (2013). BrainNet Viewer: A network visualization tool for human brain connectomics. *PLoS One* 8:e68910. 10.1371/journal.pone.0068910 23861951 PMC3701683

[B45] Zeng K. Wang Y. Ouyang G. Bian Z. Wang L. Li X. (2015). Complex network analysis of resting state EEG in amnestic mild cognitive impairment patients with type 2 diabetes. *Front. Comput. Neurosci.* 9:133. 10.3389/fncom.2015.00133 26578946 PMC4624867

