# Supplementary material for: Functional brain changes using electroencephalography after a 24-week multidomain intervention program to prevent dementia
Source: Front Aging Neurosci. 2022 Oct 12;14:892590. doi: 10.3389/fnagi.2022.892590 (PMC9597498; doi:10.3389/fnagi.2022.892590)
Supplement: Supplementary file 1 [file Data_Sheet_1.docx]

Supplementary Material

# Supplementary Tables

# Supplementary Table 1. Clinical characteristics of participants with or without EEG analysis

|  | EEG analysis  (N=127) | Non-EEG analysis  (N=25) | *p-*value |
| --- | --- | --- | --- |
| Age at baseline, years | 70.8 ± 5.0 | 72.6 ± 3.6 | 0.085 |
| Number of women | 95 (74.8%) | 18 (72.0%) | 0.769 |
| Education, years | 10.0 ± 4.8 | 10.9 ± 5.3 | 0.400 |
| Systolic blood pressure, mmHg | 125.8 ± 14.1 | 123.9 ± 13.6 | 0.543 |
| Diastolic blood pressure, mmHg | 73.4 ± 9.2 | 72.5 ± 7.8 | 0.639 |
| Hemoglobin A1c | 5.8 (5.5-6.0) | 5.8 (5.4-6.8) | 0.606^*^ |
| Dyslipidemia | 81 (63.8%) | 15 (60.0%) | 0.720 |
| Body mass index, kg/m^2^ | 24.4 ± 2.8 | 23.9 ± 2.6 | 0.399 |
| Current smokers | 3 (2.4%) | 1 (4.0%) | 0.516 |
| Current alcohol drinkers | 30 (23.6%) | 4 (16.0%) | 0.403 |
| Mild cognitive impairment | 34 (26.8%) | 9 (36.0%) | 0.349 |
| Apolipoprotein E ɛ4 carrier | 24 (18.9%) | 2 (8.0%) | 0.252 |
| Korean Mini-Mental State Examination | 27.9 ± 2.0 | 27.1 ± 2.4 | 0.089 |
| Geriatric Depression Scale-15 items | 4.2 ± 3.7 | 4.1 ± 4.5 | 0.846 |

# Values are shown as the mean ± SD, median (IQR), or n (%). *P*-value was calculated from the student t-test for the numerical data or the chi-square test or Fisher’s exact test for the categorical data. *Mann-Whitney U test. FMI, facility-based multidomain intervention; HMI, Home-based multidomain intervention.

# Supplementary Figures

(A)


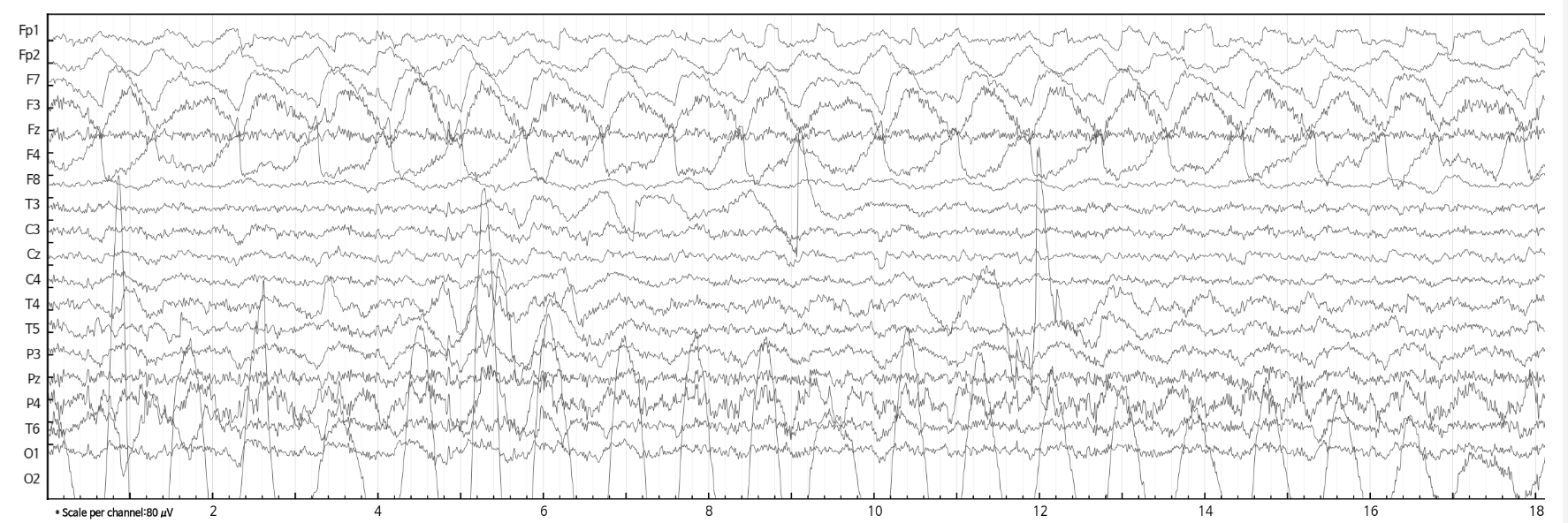


(B)

**
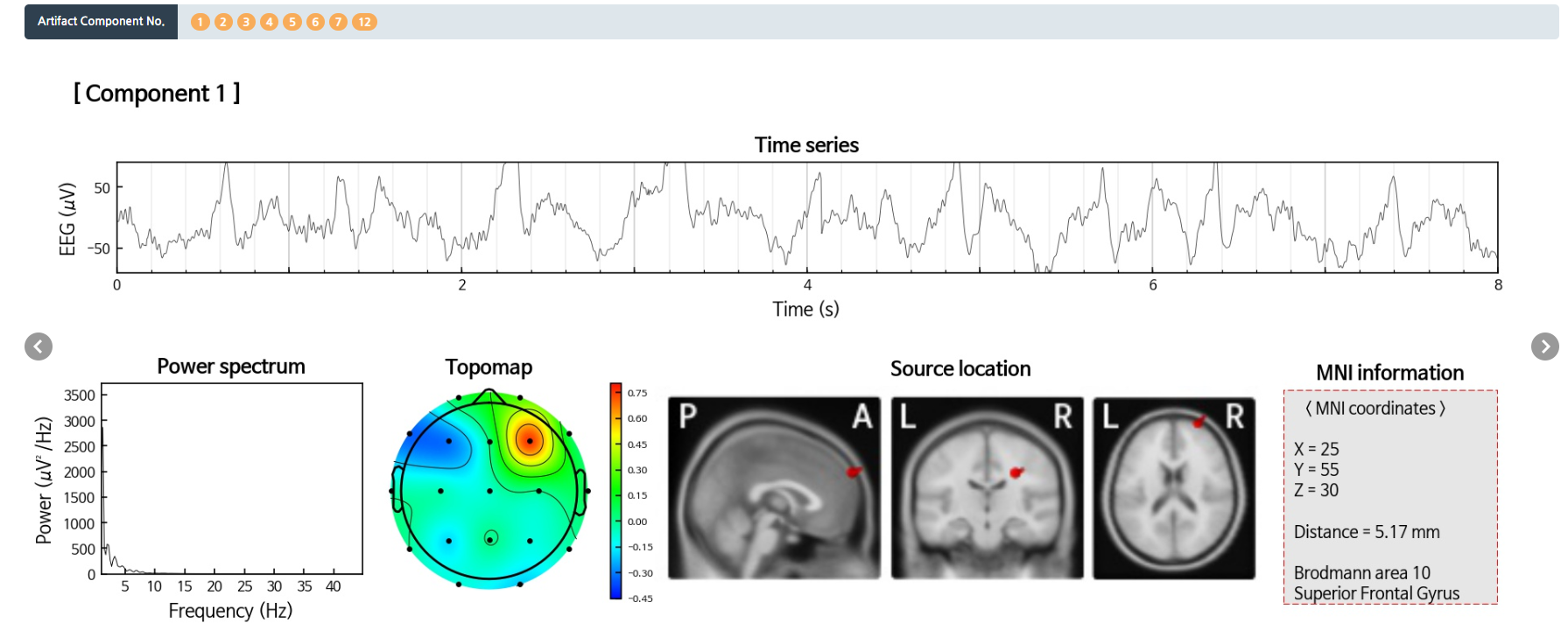
**

**
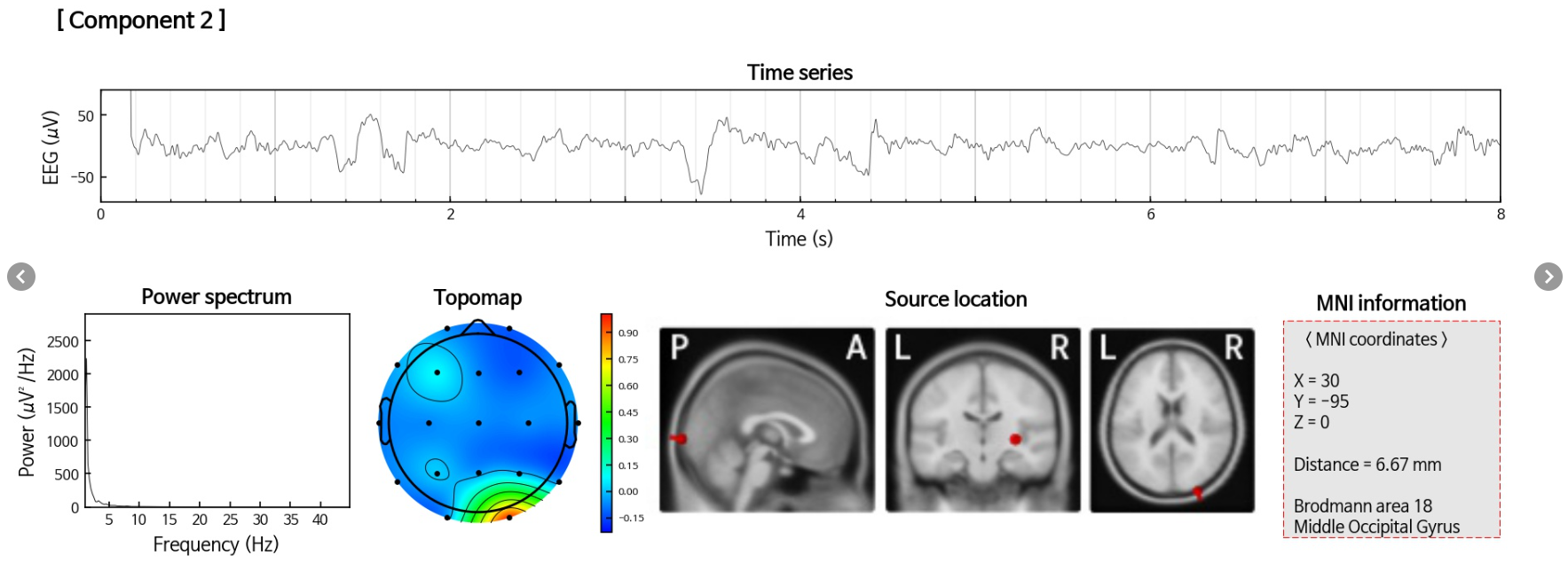
**

**
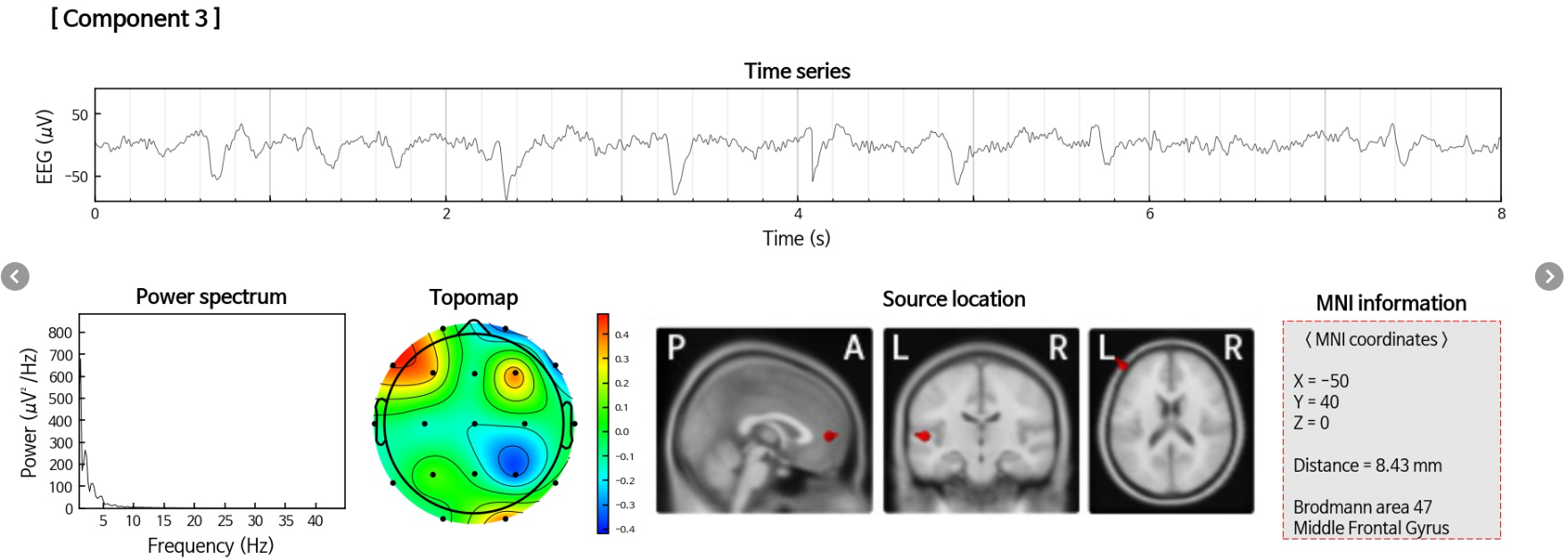
**

**Supplementary Figure 1. Example of bad quality EEG data excluded from analysis**

1. Raw data time series (linked-ear reference) while eye closed. The recorded raw data of excluded case had serious artifacts. It was difficult to think of normal brain waves after we examine it through visual inspection and independent component analysis (ICA). It can be shown that there is noise overall in the data. The overall noise cannot be removed by a denoising system using ICA. Due to most components affected by noise, it is difficult to interpret. Cardiac signal component and channel noise are found in overall frontal and occipital areas. EEG bursting is in temporal areas (channel T3 and T4)
2. Result of ICA analysis. The components from 1 to 7 were classified as the artifact components. Most of the main components were classified as artifact components. Therefore, this data is difficult to regard as normal brain waves even if reconstructed except for the artifact component.


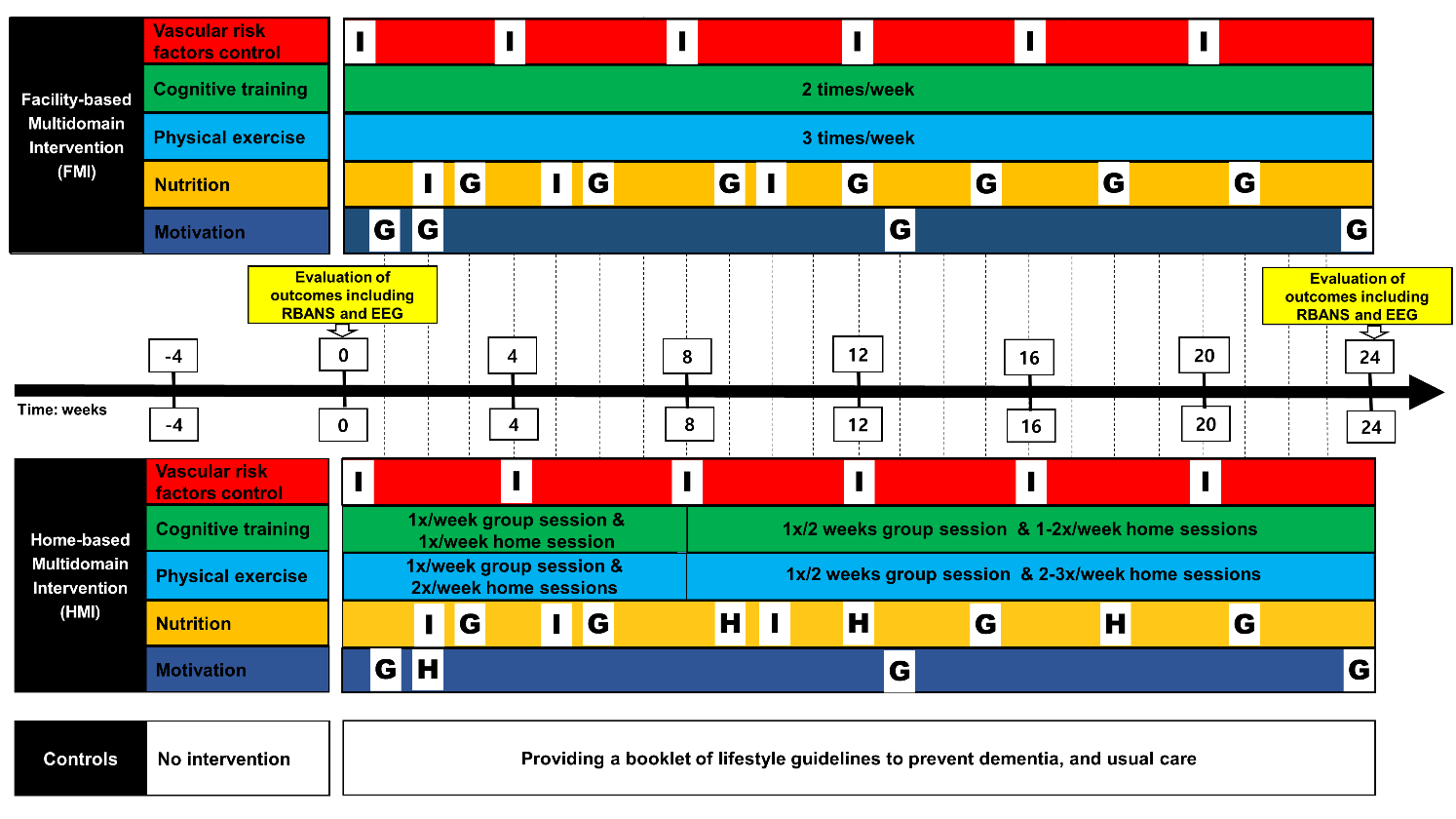


**Supplementary Figure 2.** The SoUth Korean study to PrEvent cognitive impaiRment and protect BRAIN health through lifestyle intervention in at-risk elderly people (SUPERBRAIN) protocol (Park, H.K., et al*., J. Clin. Neurol.* 2020). Cognitive training was performed twice weekly and physical exercise was performed three times weekly in the facility-based multidomain intervention (FMI) and home-based multidomain intervention (HMI) groups, while management of vascular risk factors, nutritional education, and motivational enhancement programs were offered several times according to the schedules shown. All outcome measures including the Repeatable Battery for the Assessment of Neuropsychological Status (RBANS) and electroencephalography (EEG) were performed at baseline and at the end of the study. G: group intervention, H: home-based session, I: individual intervention.
